# Comment on Chu et al. Acetaminophen’s Role in Autism and ADHD: A Mitochondrial Perspective. *Int. J. Mol. Sci.* 2025, *26*, 8585

**DOI:** 10.3390/ijms262411966

**Published:** 2025-12-12

**Authors:** Keith Fluegge, Kyle Fluegge

**Affiliations:** Institute of Health and Environmental Research (IHER), Columbus, OH 43216, USA; kyle.fluegge@gmail.com

The United States Food and Drug Administration (FDA) has now linked acetaminophen use in pregnancy to risk of offspring autism and has recommended leucovorin as a possible therapeutic for ASD [1]. This is a controversial move given recent observational evidence suggesting an inconsistent effect of acetaminophen use during pregnancy and risk of offspring neurodevelopmental disability, including autism spectrum disorder (ASD) [2,3].

Chu et al. [2] have recently published a review suggesting a theoretical framework of maternal acetaminophen use-induced mitochondrial damage in utero. In the review, the authors described the results of a series of observational studies investigating in utero exposure to acetaminophen and risk of offspring neurodevelopmental disorders. These studies have often been plagued with methodological concerns about self-reported use, imprecise dosage quantification, and the overuse of questionnaire-based methodologies, yielding inconsistent results and, therefore, no widespread change to the medical guidelines for the use of acetaminophen. Murine studies revealed that embryonic exposure to acetaminophen increased anxiety-like vocalizing behavior and reduced ambulation, while post-natal exposure reduced maze completion in adults, delayed habituation, and reduced pain thresholds. Moreover, while the authors do elaborate on the role of oxidative stress and mitochondrial abnormality in ADHD and ASD, the evidence that acetaminophen specifically induces these abnormalities or any core phenotypic expression of ASD/ADHD is rather underwhelming, especially given that the majority of ingested acetaminophen is metabolized and harmlessly excreted.

We have two main critiques of the authors’ review: Firstly, the review acknowledges that acetaminophen first entered the market in 1956, more than a decade after infantile ASD was first described. This temporal fact considerably weakens the argument of causation. Indeed, acetaminophen may be a surrogate indicator to the real cause of ASD, as is the main point of our commentary. Secondly, many of the described neurobehavioral and biochemical toxicological findings, while evident, were non-descript or occurred from drug administration above the recommended human dose equivalent and did not constitute part of the core diagnostic criterion of common neurodevelopmental pathology.

Given the policy recommendations that have been announced by the FDA, our organization thought it would be important, albeit belated, to convey our ongoing research about a potential significant environmental cause of ASD and how this factor may compel maternal acetaminophen use during pregnancy. The purpose of our commentary is three-fold.

- Introduce our proposed environmental trigger for neurodevelopmental pathology using data from our prior epidemiological investigations;
- Explain the mechanism characterizing the potential confounding role of our variable of interest;
- And, unlike Chu et al. [2], highlight how our variable displays temporal precedence with the first description of infantile ASD in 1943.

## 1. What Is the Environmental Trigger of Neurodevelopmental Pathology?

IHER has recently published a commentary with original data analysis [4], supporting our contention that ASD is a disorder of opioid dysregulation that is specifically induced from environmental emissions of nitrous oxide (N_2_O). N_2_O is well-known as a potent greenhouse gas, with a warming potential 300 times greater than that of carbon dioxide. The compound is widely used in medicine and dentistry as an analgesic, as it is thought to carry little to no harm in the acute medical setting. The compound is not metabolized by the human body, which is why medical scavenging units are critical during administration of the gas. Elevations in striatal dopamine release characterize the acute exposure effects of N_2_O, supporting its clinical use as a therapeutic for patients experiencing treatment-resistant depression and validating its public perception as a “laughing gas”.

However, N_2_O also exists in the environment as an air pollutant, derived predominately from synthetic nitrogen application in agricultural soil management. Exposure to this pollutant likely confounds the role of pesticides, and particularly glyphosate, in ASD risk. Our prior research shows a significantly positive, county-level association between glyphosate and farm use of synthetic nitrogen fertilizers [5]. Glyphosate impairs nitrogen fixation in soil through chelation of critical minerals, like manganese, necessitating the use of synthetic nitrogen in agriculture.

Unlike glyphosate and other pesticides, however, trace levels of exposure to N_2_O induce cognitive and attention deficits in healthy human subjects [6] and alter key striatal dopaminergic neurotransmission in a preclinical model of chronic, intermittent exposure [7] through putative release of dynorphin opioid peptides and activation of the kappa opioid receptor (KOR) [8]. These clinical and neurobiological effects are consistent with an ADHD phenotype and explain the therapeutic role of psychostimulants in restoring dopaminergic signaling within the limbic system. Consistently, our prior investigations link farm use of synthetic nitrogen, as the greatest source of environmental N_2_O pollution, to increased healthcare utilization for ADHD diagnosis (representing a severely impaired phenotype) in a U.S.-based cohort [9].

Conversely, farm use of synthetic nitrogen reduces hospitalization risk for autistic disorder diagnosis in the same healthcare seeking U.S. population [4]. These two-way (state and year) fixed effects regression analyses accounted for healthcare utilization for autistic disorder in HCUP states over nearly a decade, during the aftermath of improved diagnostic recognition (DSM-IV in 1994) [10] and expanded data collection efforts and awareness with the establishment of the CDCs Autism and Developmental Disabilities Monitoring (ADDM) network in 2000 [11]. These population-based data suggest, but do not prove, that, unlike ADHD, gestational exposure to environmental sources of N_2_O rewires ASD brain during critical periods of brain development, revealing an opioid dependence. Genetic studies show a comprehensive autistic syndrome in µ-opioid receptor null mice [12]. We, therefore, suspect that a heightened KOR system, an antagonistic opioid receptor subtype implicated in negative stress adaptation, socio-communicative deficits (a key diagnostic feature in ASD), drug-seeking and a strong negative regulator of brain catecholaminergic activity, then manifests in ASD brain to adapt to a lifetime exposure of chronic N_2_O emissions [4]. In addition to the manifold biological links, various climate factors have also been implicated in ASD risk, including precipitation, ozone, particulate matter, temperature, etc., which can all be explained by environmental N_2_O pollution [4].

## 2. The Mechanism of Potential Confounding

As it relates to the role of maternal acetaminophen use in pregnancy and risk of offspring ASD, it may be prudent to consider how chronic pain tolerance develops in human subjects exposed to N_2_O. The magnitude of analgesic effect from 35% N_2_O had decreased by almost half and more than a third for detection and pain thresholds, respectively, after six exposures, compared to the first N_2_O exposure [13]. Use of acetaminophen can be part of a multi-modal pain management technique for patients who have developed a tolerance to opioids [14].

**Figure 1:**
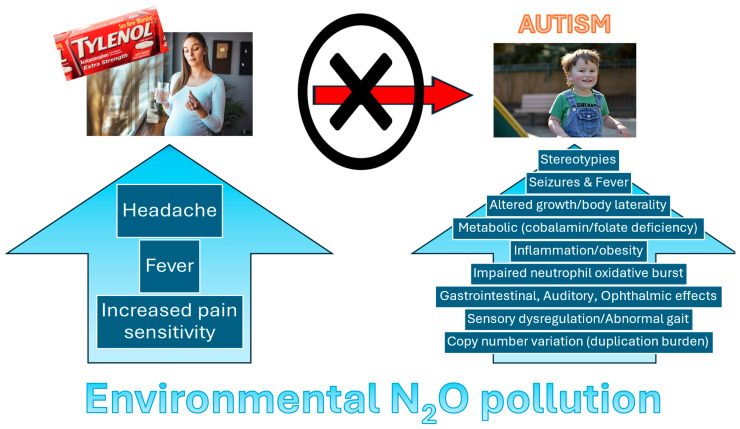
IHER proposes that environmental emissions of N_2_O, primarily from synthetic nitrogen fertilizer use in agriculture, confound the relationship between maternal use of acetaminophen during pregnancy and risk of offspring neurodevelopmental disorders, including ASD and ADHD. Chronic and/or prolonged exposure to N_2_O induces pain tolerance [13], headache [15], and fever [16] in patient populations, conditions for which acetaminophen use is clinically indicated. Moreover, preclinical studies suggest that withdrawal from trace N_2_O exposure induces stereotypic behavior [17], a key diagnostic feature of ASD, and seizure generation [18], while gestational exposure alters embryonic growth [19] and body laterality [20]. N_2_O exposure also impairs the oxidative function of neutrophils [21], which suggests that overproduction of oxidative species in ASD represents an adaptive immunophenotype and a main driver of mitochondrial dysfunction, impaired calcium regulation, etc. [22]. Consistently, we have also argued that N_2_O-induced, dynorphinergic-mediated inhibition of the alpha 7 nicotinic acetylcholine receptor [23], a ligand-gated ion channel found in both neuronal and immune cell populations that inhibits inflammation upon activation by nicotine or acetylcholine, initiates a lifespan of neurodegeneration (ADHD → Alzheimer’s disease [24,25]) but that a heightened KOR tone in ASD is protective against this neurodegeneration risk [26]. The suggestion of a neuroprotective effect is supported with evidence showing that the prevalence of Alzheimer’s disease at time of death was actually lower among those diagnosed with ASD between 1999 and 2015 compared to the general population when adjusting for changing prevalence rates [27]. Neuronal damage in animal brain from N_2_O is accompanied by behavioral alterations, hyperhomocysteinemia, and oxidative stress [28]. Clinical and preclinical effects of N_2_O upon gastrointestinal distension [29], middle ear pressure [30], eye saccades [31], feed intake [32], and sensory functioning [33] are all mirrored in ASD and likely reflect enhanced KOR tone. Additionally, comparative studies in plants suggest that N_2_O is an effective agent used for chromosomal doubling [34]. The relatedness of this finding to duplication burden in ASD [35] is unknown.

Therefore, any implication of maternal acetaminophen use in offspring ASD risk should explicitly consider how chronic environmental N_2_O exposures may be driving opioid tolerance/fever/headache among pregnant women (conditions for which acetaminophen use is clinically indicated) and thereby increase use of other pain management therapies, like acetaminophen (Figure 1). Moreover, given the long-standing, cumulative evidence from independent studies about how trace N_2_O exposure explains the core ASD symptomatology (Figure 1), we believe that environmental emissions of N_2_O are a confounding factor in the relationship between maternal acetaminophen use during pregnancy and risk of offspring neurodevelopment pathology, including ASD. Even more noteworthy, leucovorin, now being suggested as a possible approved therapeutic for ASD, has also been clinically shown to reverse the hematologic depression induced from N_2_O, presumably through the putative restoration of folate metabolism, as we have highlighted previously [4].

## 3. Temporal Precedence

**Figure 2:**
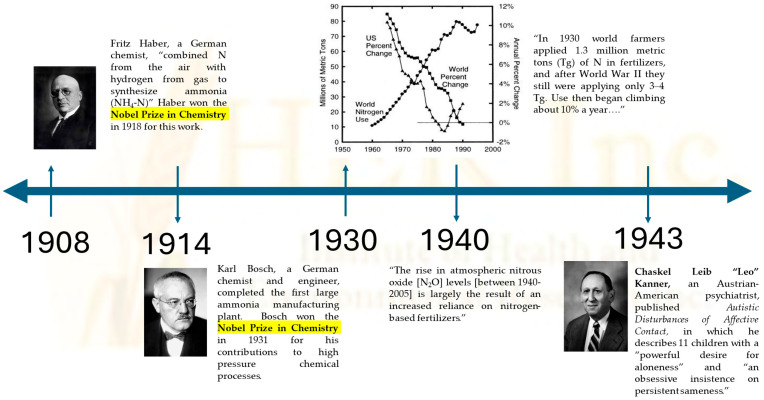
A historical timeline depicting how the adoption of synthetic nitrogen fertilizers in agriculture, derived from the Nobel prize-winning scientific work of Fritz Haber and Karl Bosch [37], directly precedes Leo Kanner’s first published case series of infantile autism in 1943 [38]. Farm use of synthetic nitrogen fertilizers is the greatest source of environmental N_2_O emissions [36]. Given the clinical, preclinical, and comparative analysis studies as well as the temporal precedence, IHER believes that environmental N_2_O emissions, even at trace levels, induce the signature phenotypic manifestations of common neurodevelopmental disorders. Our longitudinal fixed effects regression analyses paint a disturbing reality that celebrated advances in industrialized food production have contributed significantly to real and costly public health burdens, including not only ASD [4,8,26] and ADHD [5,9,24,25] but neurodegeneration [24,25,26] and other comorbidities.

Farm use of synthetic nitrogen fertilizers is widely recognized as the most significant contributor to environmental N_2_O emissions since 1940 [36]. The introduction of synthetic nitrogen in agriculture occurred early in the preceding decade [37], while Leo Kanner’s first reported case series of ASD in children was published in 1943 [38]. The chronological order of these events is consistent with our hypothesis that rising N_2_O emissions from an increasingly industrialized agricultural system cause neurodevelopmental abnormality (Figure 2), demonstrating temporal precedence. Interestingly, world use of synthetic nitrogen peaked in 1988, as shown in Figure 2 [37]. This time period coincides with the year that HHS Secretary RFK Jr. has cited as when an unknown environmental toxin triggered the worrisome rise in prevalence of neurodevelopmental disorders.

## 4. Future Directions

To further our prior epidemiological investigations, IHER has begun a program of individual assessment of chronic environmental exposure to N_2_O. Preliminary assessments from direct combustion sources indicate a level of exposure that matches or exceeds levels that have been shown to clinically alter human cognitive functioning and neurotransmission in animal models of N_2_O neurotoxicity (≥50 ppm). Future comprehensive assessments should be conducted in at-risk populations (i.e., those diagnosed with ADHD) and from a wide variety of environmental sources (including farmland, vehicular pollution, bodies of water, low-level elevation hotspots where environmental N_2_O emissions could be expected to pool, and even bacterial denitrification processes in human models of disease pathogenesis [39], etc.) to clearly delineate potential individual exposure to environmental emissions of N_2_O.

However, exposure to N_2_O is not recommended for pregnant women due to the known risk for reproductive harm. While the American Dental Association (ADA) recommends the use and proper maintenance of scavenging systems and setting exposure limits for occupational exposure, the ADA also specifically advises that pregnant patients should avoid it whenever possible [40]. According to NIOSH, the exposure limit is 25 ppm for exposure to N_2_O in the dental settings [41]. Worryingly, IHER’s preliminary emissions testing indicates levels of direct environmental N_2_O emissions from combustion sources to be at least more than double this estimate. Given such concern from regulatory and scientific bodies, we concur that exposing pregnant women to N_2_O to prospectively observe ASD outcomes or acetaminophen use is an unethical research question to pursue. Care must be taken to ensure the least amount of harm when investigating the link between environmental emissions of N_2_O and neurodevelopmental outcomes.

Perhaps most importantly, our novel findings support decades of independent research linking trace N_2_O exposure to all key phenotypic manifestations of ASD (Figure 1). We are not aware of any other factor that has been able to explain the core phenotype, as well as notable comorbidities, of an ASD diagnosis, and exhibit temporal precedence with the first description of infantile ASD in 1943. Therefore, we advocate additional study on the magnitude of N_2_O emissions, development of individual exposure models, and especially well-designed studies of the effects of environmental N_2_O pollution on neurodevelopmental risk. Our research also critically links known agents of global climate warming to direct human health costs and presents a stark, perhaps inconvenient, reality check to the scientific community.

### Disclosure

The Institute of Health and Environmental Research (IHER) is a 501c(3) research-based non-profit organization. Both authors are IHER directors and participated in the research and the writing of the commentary. The institute has received no funding for this work and has no conflicts of interest.

The objectives of IHER are detailed below:

a. To be a not-for-profit organization:
  1. The IHER is organized exclusively for charitable, religious, educational, and scientific purposes, including, for such purposes, the making of distributions to organizations that qualify as exempt organizations under Section 501(c)3 of the Internal Revenue Code, or the corresponding section of any future federal tax code.
b. To work towards the betterment of environmental health and well-being by protecting natural ecosystems and fostering sustainable and environmentally sensitive agricultural systems.
c. To work towards the betterment of public health by prevention and control of disease and illness, thereby preventing the distress due to these now and in the future;
d. To investigate links between human and environmental health.
e. In order to fulfill these, the Institute aims
  (i) To perform and to disseminate scientific research in human and environmental health in the public interest;
  (ii) To inform and educate students and the public about issues related to human and environmental health in the public interest;
  (iii) To provide funds to individuals, bodies or institutions having similar objects to the organization that further the aims of the organization;
  (iv) To advocate the objects and policies of the organization, and for changes to practice and to international and national law to reflect research results;
  (v) To promote links between the non-profit and individuals and organizations with similar aims interstate and overseas;
  (vi) To establish and maintain a public fund to be called The Institute of Health and Environmental Research Fund for the specific purpose of supporting the objects of the non-profit.

## Data Availability

No new data were created or analyzed in this study. Data sharing is not applicable to this article.
